# Phenolic Compounds from *Humulus lupulus* as Natural Antimicrobial Products: New Weapons in the Fight against Methicillin Resistant *Staphylococcus aureus*, *Leishmania mexicana* and *Trypanosoma brucei* Strains

**DOI:** 10.3390/molecules24061024

**Published:** 2019-03-14

**Authors:** Laetitia Bocquet, Sevser Sahpaz, Natacha Bonneau, Claire Beaufay, Séverine Mahieux, Jennifer Samaillie, Vincent Roumy, Justine Jacquin, Simon Bordage, Thierry Hennebelle, Feng Chai, Joëlle Quetin-Leclercq, Christel Neut, Céline Rivière

**Affiliations:** 1 EA 7394—ICV, Charles Viollette Research Institute, SFR Condorcet FR CNRS 3417, Univ. Lille, INRA, ISA-Yncréa, Univ. Artois, Univ. Littoral Côte d’Opale, 3 rue du Professeur Laguesse, 59000 Lille, France; laetitia.bocquet1309@gmail.com (L.B.); sevser.sahpaz@univ-lille.fr (S.S.); natacha.bonneau@univ-lille.fr (N.B.); jennifer.samaillie@univ-lille.fr (J.S.); vincent.roumy@univ-lille.fr (V.R.); justine.jacquin@yncrea.fr (J.J.); simon.bordage@univ-lille.fr (S.B.); thierry.hennebelle@univ-lille.fr (T.H.); 2 Pharmacognosy Research group, Louvain Drug Research Institute, Université Catholique de Louvain, 1200 Brussels, Belgium; claire.beaufay@uclouvain.be (C.B.); joelle.leclercq@uclouvain.be (J.Q.-L.); 3 U995—LIRIC, Lille Inflammation Research International Center, Univ. Lille, Inserm, CHU Lille, 59000 Lille, France; severine.mahieux@univ-lille.fr (S.M.); christel.neut@univ-lille.fr (C.N.); 4 U1008—Controlled Drug Delivery Systems and Biomaterials, Univ. Lille, Inserm, CHU Lille, 59000 Lille, France; feng.chai@univ-lille.fr

**Keywords:** *Humulus lupulus*, prenylated phenolic compounds, antimicrobial agents, methicillin-resistant *Staphylococcus aureus*, *Leishmania mexicana mexicana*, *Trypanosoma brucei brucei*

## Abstract

New anti-infective agents are urgently needed to fight microbial resistance. Methicillin-resistant *Staphylococcus aureus* (MRSA) strains are particularly responsible for complicated pathologies that are difficult to treat due to their virulence and the formation of persistent biofilms forming a complex protecting shell. Parasitic infections caused by *Trypanosoma brucei* and *Leishmania mexicana* are also of global concern, because of the mortality due to the low number of safe and effective treatments. Female inflorescences of hop produce specialized metabolites known for their antimicrobial effects but underexploited to fight against drug-resistant microorganisms. In this study, we assessed the antimicrobial potential of phenolic compounds against MRSA clinical isolates, *T. brucei* and *L. mexicana*. By fractionation process, we purified the major prenylated chalcones and acylphloroglucinols, which were quantified by UHPLC-UV in different plant parts, showing their higher content in the active flowers extract. Their potent antibacterial action (MIC < 1 µg/mL for the most active compound) was demonstrated against MRSA strains, through kill curves, post-antibiotic effects, anti-biofilm assays and synergy studies with antibiotics. An antiparasitic activity was also shown for some purified compounds, particularly on *T. brucei* (IC_50_ < 1 to 11 µg/mL). Their cytotoxic activity was assessed both on cancer and non-cancer human cell lines.

## 1. Introduction

Multidrug-resistant microorganisms are rapidly spreading throughout the world leading to treatment failures [1]. Antibacterial resistance in humans is affected by an inappropriate or excessive use of antibiotics in human health but is also partially affected by the use of antibiotics in animal-rearing. Some national action plans, such as restrictive measures of antibiotic use in human and animal health, are now imposed in all parts of the world but the problem of antimicrobial resistance is far from being solved [2,3]. Therefore, the discovery of new antimicrobial compounds is crucial.

Several plant-derived natural products, characterized by a huge structural diversity, including phenolic compounds, are cited as antimicrobial agents and resistance-modifying agents (RMAs) [4]. They could constitute a valuable interim solution, until new classes of antibiotics are discovered [5].

Methicillin-resistant *Staphylococcus aureus* (MRSA) strains are considered as a very urgent health problem because of their propagation in the last 15 years in the elderly and in immunocompromised patients, mainly due to their increasing implication in nosocomial infections and the lack of development of new antimicrobials. In 2016, the percentage of MRSA among all *S. aureus* isolates remained above 25% in several countries in Southern Europe and greater than 15% in France, especially for invasive isolates [6]. MRSA is also one of the principal multi-resistant bacterial pathogen responsible for complicated skin and hospital-acquired infections, associated with high mortality rates [7,8]. Furthermore, these strains are very often involved in infections of diabetic foot ulcers characterized by frequent complications and risks of lower-limb amputations [9]. *S. aureus* has the ability, like most Gram-positive organisms, to acquire resistance to practically all useful antibiotics. Several mechanisms can explain this resistance: modification of target sites, enzymatic degradation or structural modification of antibiotics and, expression of efflux pumps [5,10,11,12]. These bacteria also produce virulence factors involved in pathogenesis like adhesion and biofilm formation, which enhance bacterial resistance [13,14].

On the other hand, many parasitic infections deserve a special attention due to the lack of effective treatments. *Trypanosoma brucei* is a parasite conveyed by the tsetse fly and responsible for the Human African Trypanosomiasis (HAT) or sleeping sickness. This fatal disease if untreated leads to central nervous system disturbances, including sensory, motor and psychic troubles and neuroendocrine abnormalities [15,16]. Leishmaniasis, caused by different *Leishmania* species, is responsible for chronic skin and visceral diseases transmitted by sand-flies [17].

**Figure 1:**
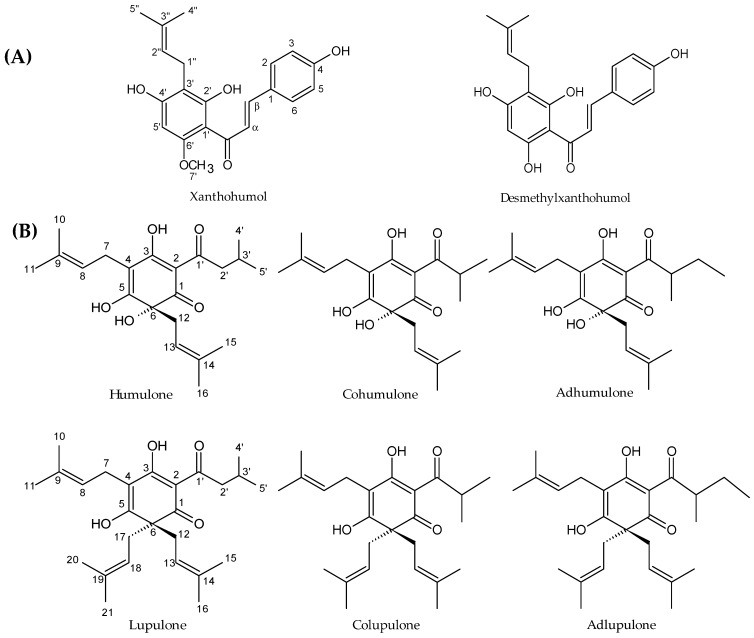
Structures of the main prenylated phenolic compounds from hops. (**A**) prenylated chalcones (**B**) acylphloroglucinol derivatives.

Hop (*Humulus lupulus* L.) is a climbing dioecious plant belonging to the Cannabaceae family. This species is cultivated worldwide for its female inflorescences (cones), usually called “hops”, which are used in the brewing industry. The compounds sought by brewers are prenylated acylphloroglucinol derivatives, also called bitter acids and in particular α-acids (humulone derivatives) which are isomerized into iso-α-acids during the brewing process. These compounds confer some bitterness and antiseptic properties to beer [18]. This plant is also known to have many pharmacological activities, including sedative, oestrogenic, anti-inflammatory, chemopreventive and antimicrobial activities [19]. The antimicrobial activity is mainly attributed to prenylated acylphloroglucinols, in particular to α-acids and β-acids (lupulone derivatives) and to prenylated chalcones, including xanthohumol (Figure 1) [20,21,22]. Few studies, however, describe the activity of this plant and especially of its specialized metabolites against multidrug resistant strains [14,23] and parasites [24,25]. In addition, the mechanisms of the inhibitory activities observed are not really expanded upon now.

In this context, we studied the antimicrobial potential of hop phenolic compounds against MRSA strains, *Trypanosoma brucei brucei* (Tbb) and *Leishmania mexicana mexicana* (Lmm). We opted for a fractionation using Centrifugal Partition Chromatography (CPC) and preparative High-Performance Liquid Chromatography (HPLC) to purify the major antimicrobial phenolic compounds from the most active sub-extract of hop. Purified compounds were then quantified in hop extracts. Their antibacterial potential against MRSA clinical isolates was assessed. The most promising compounds were then selected for further experiments in order to strengthen our knowledge of their action on the bacterial strains, through kill curves, post-antibiotic effects, anti-biofilm and synergy assays. The antiparasitic activity of some chalcones and acylphloroglucinols against Tbb and Lmm was also evaluated. Their cytotoxicity activity was determined on various human cell lines.

## 2. Results

### 2.1. Antimicrobial Activity of Hop Extracts and Sub-Extracts against Gram-Positive and Gram-Negative Bacteria and Parasites

We evaluated the antimicrobial activity of hop extracts and sub-extracts towards bacterial clinical isolates and parasites (Tbb and Lmm).

We first confirmed the antibacterial activity of the crude hydro-ethanolic extract of cones against Gram-positive bacteria, namely *Corynebacterium*, *Enterococcus*, *Mycobacterium*, *Staphylococcus* and *Streptococcus* strains, with MICs ranging from 39 to 156 µg/mL (Table 1). Some yeasts have also been studied. On the 36 *Candida albicans* strains tested (data not shown), only the two presented in Table 1 were susceptible to our extracts. Leaves, stems and rhizomes showed a weak antimicrobial activity. The liquid-liquid fractionation of the most active crude extract (cones) with dichloromethane (DCM) and water led to set up a second screening only focused on Gram-positive bacteria (Table 2). This partition also led us to direct the activity towards non-polar compounds as the non-polar sub-extract was indeed more active than the crude extract. Enterococci appeared less susceptible to the DCM sub-extract than staphylococci and streptococci. *S. aureus* strains were the most susceptible with MICs ranging from 9.8 to 19.5 µg/mL (Table 2). This non-polar fraction of hops is known to contain prenylated chalcones and acylphloroglucinol derivatives (Figure 1) which were further purified to analyze their activity.

Extracts and sub-extracts were also tested for their antiparasitic effect. This activity was for the first-time described here on Tbb with respective IC_50_ of 7.8 and 4.6 µg/mL for the hydro-ethanolic crude extract and the DCM sub-extract of cones. Hops is less active against Lmm with IC_50_ of 29.0 and 28.2 µg/mL, respectively.

### 2.2. Purification of Hops Prenylated Chalcones and Acylphloroglucinols and Quantification in Hop Samples

**Figure 2:**
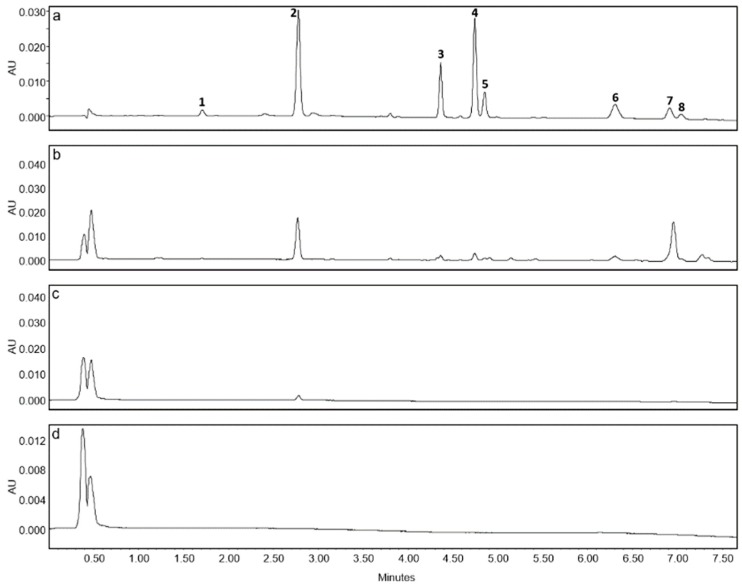
LC-UV chromatograms (370 nm) of crude extracts prepared at 100 µg/mL in MeOH for (**a**) cones and 1 mg/mL in MeOH for (**b**) leaves, (**c**) stems and (**d**) rhizomes. Compounds identified are as follows: desmethylxanthohumol (**1**) rt 1.70 min, xanthohumol (**2**) rt 2.78 min, cohumulone (**3**) rt 4.37 min, humulone (**4**) rt 4.75 min, adhumulone (**5**) rt 4.86 min, colupulone (**6**) rt 6.31 min, lupulone (**7**) rt 6.92 min, adlupulone (**8**) rt 7.05 min.

Major prenylated chalcones (xanthohumol and desmethylxanthohumol) and prenylated acylphloroglucinols (cohumulone, humulone, colupulone and lupulone) were purified from the active DCM sub-extract of cones by centrifugal partition chromatography (CPC) and preparative high-performance liquid chromatography (HPLC) (Figure 1). Their structure was established by comparison with their spectral data, including NMR data and mass spectra, with reported values [26,27,28,29] (Supplementary Material, Tables S2–S4). These compounds were identified in the different plant crude extracts by UHPLC-UV-MS on the basis of their retention times and their mass spectrum (Figure 2 and Supplementary Material, Figures S1–S3). They were then used for quantification in different parts of the plant and for activities testing.

Quantification was performed on crude hydro-ethanolic extracts of the cultivar ‘Nugget’ used for bioassays. Concerning our method, good linearity was observed for each compound over the concentration range (Supplementary Material, Table S1). Evaluation of the method on the cones extract showed acceptable intra- and inter-day precisions for xanthohumol (RSD% = 10.6, 12.0), humulone (RSD% = 12.4, 13.8) and lupulone (RSD% = 10.3, 10.7).

**Figure 3:**
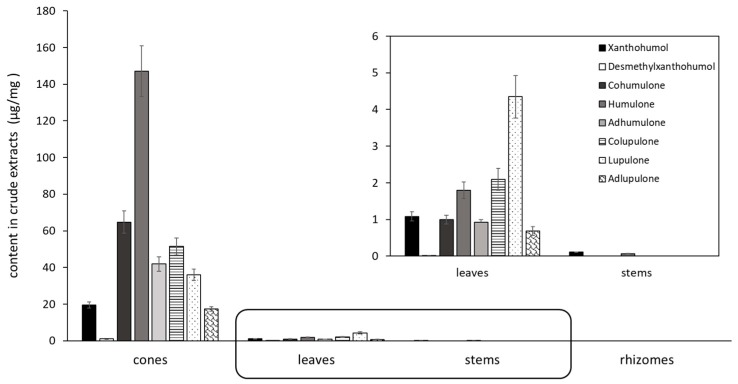
Content of prenylated chalcones and acylphloroglucinols in crude hydro-alcoholic extracts of different hop parts (in µg/mg) (*n* = 3, mean ± SD).

The contents in prenylated phenolic compounds were much higher in the crude extract of cones than in stems, roots and leaves crude extracts (Figure 2 and Figure 3). Only xanthohumol and humulone have been quantified in the stems extract. No prenylated phenolic compounds could be quantified in the rhizome extract. Interestingly, humulone was the main compound in cones extract (147 µg/mg), with a total alpha/beta acid (humulone/lupulone derivatives) ratio of 2.4, whereas lupulone was the most abundant compound in leaves extract, with a ratio of 0.5.

### 2.3. Antibacterial Activity of Purified Prenylated Phenolic Compounds

#### 2.3.1. Antibacterial Activity of Purified Compounds against Selected MRSA and Methicillin-Sensitive *Staphylococcus aureus* (MSSA) Strains

All compounds tested were active against selected oxacillin susceptible and resistant *S. aureus* clinical isolates (Table 3). Indeed, methicillin resistance is routine researched by using oxacillin disks. The first strain (T28.1) was more intensively studied because it combined many pathologic features like isolation from a diabetic foot infection and harbouring genes implicated in antibiotic resistance, capsule formation and biofilm formation [30]. Lupulone was the most promising compound with MICs from 0.6 to 1.2 µg/mL towards MRSA strains. With the exception of lupulone, we demonstrated that xanthohumol and desmethylxanthohumol were more active than bitter acids, with MICs ranging from 9.8 to 19.5 and 19.5 to 39 µg/mL towards MRSA. However, desmethylxanthohumol was slightly less active than xanthohumol. MICs did not differ between MRSA and MSSA strains.

**Figure 4:**
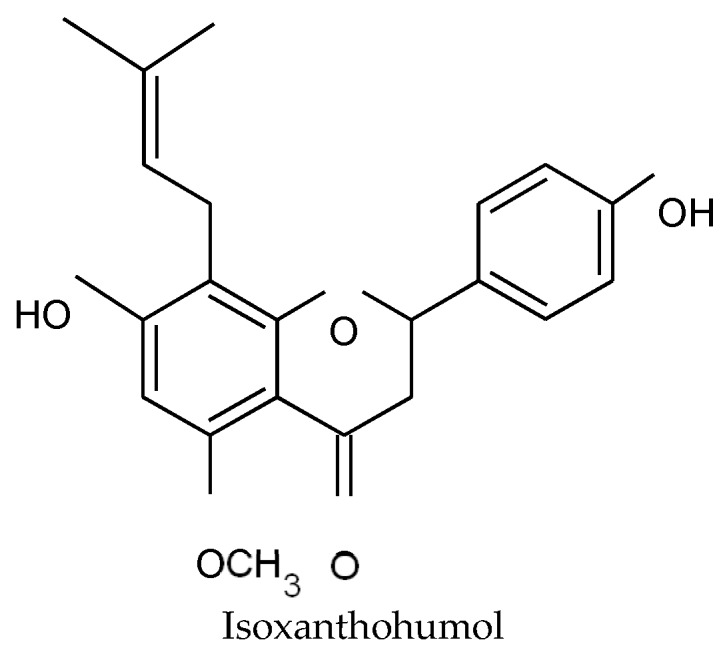
Structure of isoxanthohumol.

Isoxanthohumol (Figure 4), a flavanone known to be a metabolite of xanthohumol, was also tested against the strain MRSA T28.1 and its MIC was found to be of similar activity as desmethylxanthohumol (MIC = 39 µg/mL).

Xanthohumol, desmethylxanthohumol and lupulone were selected for further experiments on the *S. aureus* T28.1 clinical isolate, in order to better understand how they act on MRSA.

#### 2.3.2. Further Experiments on MRSA T28.1 with the Most Promising Compounds Xanthohumol, Desmethylxanthohumol and Lupulone

##### Synergies Assessed by Checkerboard

One of the strategies employed to overcome resistant strains is the combination of current antibacterial agents with natural compounds. Hops phenolic compounds and selected antibiotics (ciprofloxacin, gentamicin, oxacillin and rifampicin) were combined using the checkerboard method, to establish the best combination of products for increasing the activity [32].

The antibacterial action of hops compounds can be enhanced by combining xanthohumol with desmethylxanthohumol or with lupulone, showing an additive effect (Table 4). In contrast, the combination of desmethylxanthohumol with lupulone was found to be antagonistic.

The combination of hops compounds with selected antibiotics highlighted interesting effects (Table 4). Xanthohumol was synergistic to additive with the four antibiotics. For example, rifampicin can be 8 times more active when combined with xanthohumol. Desmethylxanthohumol showed a synergistic activity with gentamicin, synergistic to additive with ciprofloxacin and additive with oxacillin, while sometimes antagonist effect was observed with rifampicin. The interaction of lupulone with ciprofloxacin was additive and this association was antagonist or indifferent with gentamicin and rifampicin.

##### Kill Curve

**Figure 5:**
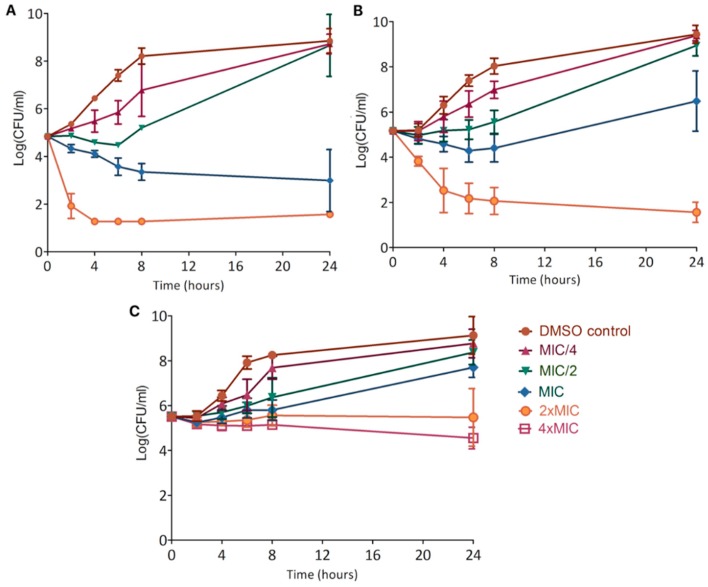
Kill curves showing the effect of xanthohumol (**A**), desmethylxanthohumol (**B**) and lupulone (**C**) on *S. aureus* T28.1 growing during 24 h. The detection threshold of the experiment is 1 log(CFU/mL).

Kill curves allowed the determination of time-dependent bacteriostatic and bactericidal concentrations of each product [33] (Figure 5).

Xanthohumol decreased the bacterial population by 0.5 log(CFU/mL) during the first 6 h at sub-inhibitory concentration (MIC/2) and by 2 log(CFU/mL) during 24 h at the MIC. It was bactericidal at 2xMIC, rapidly decreasing the bacterial population to around 3 log(CFU/mL) during the first 4 h which means that only 1 bacterium among 1000 initially present is still alive (Figure 5A).

Desmethylxanthohumol decreased the bacterial population during the first hours at the MIC. The bacteria grew back after 6 h of culture. However, it was bactericidal at 2 × MIC; reaching the detection threshold of 1 log(CFU/mL) after 24 h (Figure 5B).

Although lupulone was the most active compound (based on MIC of 1.2 µg/mL), it slightly decreased the bacterial population only at 4 × MIC. Lower concentrations just slowed down growth (Figure 5C) and a reduction between the control and 2 × MIC is about 3 log(CFU/mL).

##### Post-Antibiotic Effects

**Figure 6:**
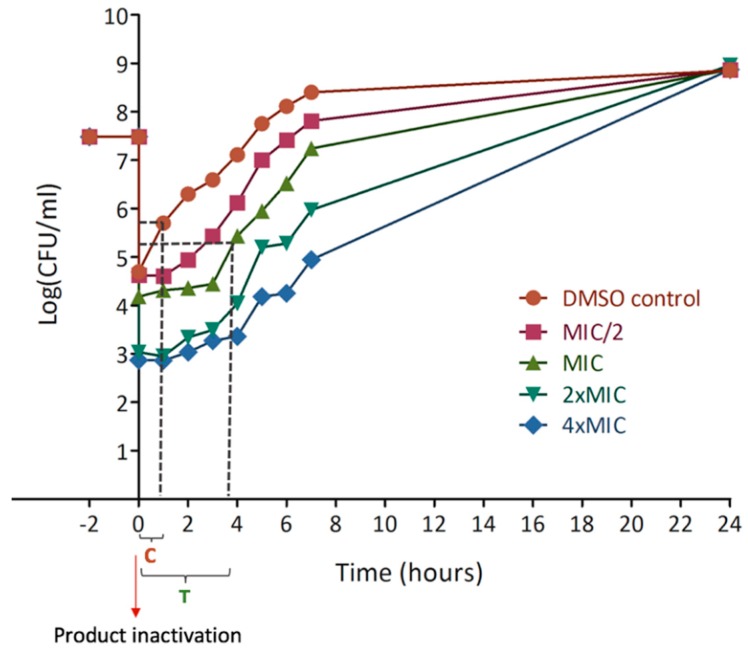
Post-antibiotic curve of xanthohumol indicating graphical determination of parameters for the determination of the growth delay. Time between −2 and 0 shows the pre-treatment with antibacterial product. Time 0 corresponds to the product inactivation. The growth retardation is quantified during the first hours of culture, comparing each condition with the control, using the formula: PAE = T − C. Where T is the time needed for the bacterial population to grow by 1 log10 (the present example is for the MIC) and C is the corresponding time for the control.

Post-antibiotic tests highlighted the fragility of the bacterial population after 2 h exposure to the active compounds followed by inactivation, which resulted in a delayed regrowth [33]. The growth retardation is quantified by the difference between the time necessary for each condition to grow from a log10 (T) and the corresponding time for the control (C). These parameters are graphically determined as shown in Figure 6.

The three compounds tested caused delayed growth at all concentrations evaluated (Table 5).

Here again, even if lupulone was the most active (based on MIC values), it caused a low effect after its inactivation with a maximum delay close to 55 min. In contrast, xanthohumol and desmethylxanthohumol caused a significant delay for regrowth. Desmethylxanthohumol inhibited bacterial regrowth for up to almost 2.5 h after pre-treatment at the MIC. Xanthohumol had the same effect at the MIC and it increased with the concentration, the delay reaching 3.29 h at 4 × MIC.

##### Anti-Biofilm Assays

Biofilms contribute to bacterial resistance, forming a complex protecting shell [34]. We initially considered the activity of hops compounds on artificial surfaces, which was confirmed on a bone substitute, the natural colonized substrate of the model strain *S. aureus* T28.1. The influence of the compounds was assessed both on the biofilm formation and on the destruction of preformed biofilms.

**Figure 7:**
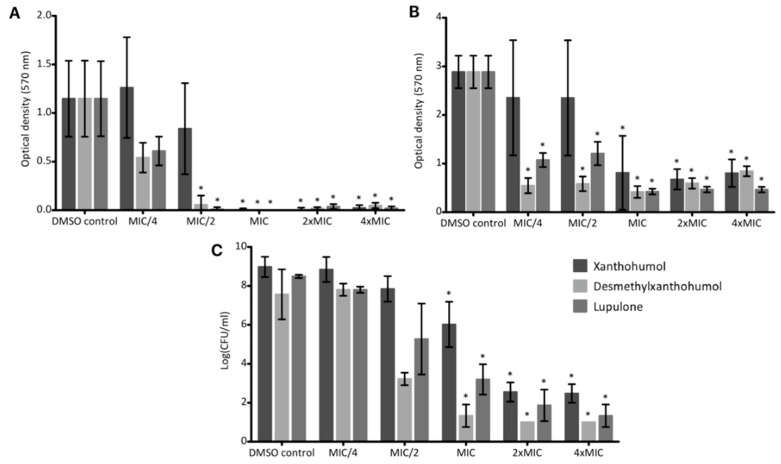
Effect of xanthohumol, desmethylxanthohumol and lupulone on the biofilm formation (**A**) of *S. aureus* T28.1 on artificial surface (**A**), on the biofilm destruction of *S. aureus* T28.1 on artificial surface (**B**) and on the biofilm formation of *S. aureus* T28.1 on bone substitute (**C**). According to Shapiro test, means tagged with * are significantly different from the control (*p* = 0.05) using Kruskal Wallis and Dunn’s tests for (**A**) and for lupulone and desmethylxanthohumol for (**C**) or ANOVA and Tukey test for (**B**) and for xanthohumol for (**C**).

Xanthohumol totally inhibited the biofilm formation on artificial surface at the MIC (Figure 7A), which correlates with the bactericidal effect pointed out with kill curves (Figure 5A). Desmethylxanthohumol and lupulone showed a significant inhibition of biofilm formation at sub-inhibitory concentrations (Figure 7A). Thus, this effect seems independent of the bactericidal activity (Figure 5B,C). The same trend was observed on bone substitutes (Figure 7C). Even if the inhibition of the biofilm formation is found less intense in bone substitute, this potential remains very interesting, in particular for desmethylxanthohumol and lupulone. These last products showed a significant decrease of the biofilm formation on bone substitute discs at MIC (Figure 7C).

Hops compounds are also able to destroy the biofilm. Xanthohumol showed a non-dose-dependent activity at the MIC and above, which is similar to the inhibition dose for the biofilm formation (Figure 7B). As seen previously, desmethylxanthohumol and lupulone showed a greater anti-biofilm potential than xanthohumol. A significant biofilm destruction was also observed at sub-inhibitory concentrations, at MIC/4, with 81% and 62.8%, respectively.

### 2.4. Antiparasitic Activity of Purified Prenylated Phenolic Compounds

The two chalcones, xanthohumol and desmethylxanthohumol, as well as two acylphloroglucinols, humulone and lupulone, were tested against the two parasites: *Trypanosoma brucei brucei* (Tbb) and *Leishmania mexicana mexicana* (Lmm) and were active. Lupulone is the most promising compound with IC_50_ of 0.9 and 4.7 µg/mL against Tbb and Lmm, respectively (Table 6). As for antibacterial activities, we demonstrated that xanthohumol and desmethylxanthohumol were more active than humulone on parasites, with IC_50_ from 2.4 to 6.1 and 7.7 to 26.2 µg/mL on both Tbb and Lmm.

### 2.5. Cytotoxicity

We evaluated the antiproliferative activity of hops compounds on various cancer and non-cancer cell lines (Table 7). We showed that desmethylxanthohumol is the less toxic compound. Considering its moderate activity, active dose could be toxic. Active concentration of desmethylxanthohumol on Tbb is the only one lower than the cytotoxicity on the tested cell lines. Xanthohumol was also toxic at the antibacterial concentration on all the targeted cell lines. For both chalcones, selectivity indexes (cytotoxic IC_50_/active IC_50_) would be close to 1. A special attention has to be paid on lupulone, its pronounced antibacterial activity gives selectivity index > 4 compared to its cytotoxicity against MG-63 (Table 7). Antibacterial and anti-biofilm concentrations showed a cell viability close to 100%. These data make lupulone a very good candidate for a topical bone application.

## 3. Discussion

Multi-drug resistance (MDR) bacteria, like methicillin-resistant *Staphylococcus aureus* (MRSA), present a major challenge for the medical community in the treatment of infections, such as diabetic foot infections [6,35]. The discovery of new antibiotics is not fast enough to offset the global spread of resistant pathogens. At the same time, we can observe a worrying emergence of resistance to some of the newer antibiotic agents [36,37,38]. Hence, the development of combination of current agents with other type of resistance-modifying agents, such as natural antibacterial agents, can be an alternative strategy to overcome MDR [39].

In this context, we evaluated the antibacterial potential of hop extracts and more particularly of three pure compounds isolated from hops (xanthohumol, desmethylxanthohumol and lupulone) towards MRSA, one of the most aggressive agents, with different approaches [33]. We began by a classical MIC determination, which is only an endpoint method, followed by kill-time curves assessing the time dependent effect and synergistic studies, in combination with antibiotics, giving more information on their resistance modifying potential. The checkerboard method is the preferred technique of choice to analyze these interactions, with however a certain variability in the methods of interpretation [32]. The study of a post-antibiotic effect (PAE), also applicable to substances other than antibiotics, made it possible to follow the regrowth of bacteria after inactivation of the antibacterial substance after a defined contact time at an active concentration. When damaged bacteria need some time to recover in comparison to the control, the regrowth will be lowered, which means in clinical situations that administration intervals of the antimicrobial substances can be delayed. Another innovative point is the effect on biofilms, concerning about 60% of infections. In biofilms, the bacteria are surrounded by a thick layer of extracellular polysaccharides which makes them inaccessible to antibiotics but also to defenses of the immune system. Furthermore, the lack of nutrients lowers dramatically the bacterial multiplication. As most of the antibiotics act on mechanisms implicated in multiplication, this is the second reason for their lack of action on biofilms. Influence on biofilms can be studied in two manners: destruction of established biofilms or inhibition of their formation. The first manner is more relevant for clinicians as at the time of diagnosis the biofilm is already established.

The antibacterial activity of hops has been known for many years [40,41] but has been poorly evaluated against resistant strains. We showed here their effects on *Corynebacterium*, *Enterococcus*, *Mycobacterium*, *Staphylococcus* and *Streptococcus* strains, some being resistant to antibiotics (Table 1). Their effect on *Bacillus*, *Streptomyces* and *Micrococcus* strains was previously underlined [22,41]. Hops extracts are also able to combat some human pathogenic bacteria found in food, such as *Clostridium perfringens* and *Listeria monocytogenes* [42,43]. The screening conducted on some *C. albicans* strains showed that the spectrum of activity of hops extracts against these yeasts is weak, only two strains were susceptible to our extracts. The *C. albicans* ATCC 10,231 reference strain was previously tested by Langezaal et al. [44] who also found an efficiency of cones extract. Other studies have demonstrated that hops extracts are more efficient on bacteria than yeasts, showing no effect of hops constituents on *Saccharomyces* strains [41]. Contrarily to Langezaal et al. [44] and Abram et al. [45] who have detected a slight activity of cones extracts against *E. coli* strains, our extracts were inactive towards Gram-negative bacteria. Stems, leaves and rhizomes have been rarely studied. According to our results, their crude hydro-ethanolic extracts showed a weak antimicrobial activity in comparison with cones, as already highlighted for leaves against some bacteria [45]. Nevertheless, the rhizomes extract appeared to be more active than leaves and stems extracts (Table 1). Previous research on the quantification of the phenolic hop compounds in cones and leaves by LC-UV is reported in the literature [46]. With our method of quantification, no prenylated phenolic compounds could be quantified in the rhizome extract. Consequently, the activity of rhizomes could be related to the presence of other metabolites. The antibacterial activity of cones was mainly attributed to apolar compounds because the DCM sub-extract of cones was more active than the crude extract of the same plant part (Table 2). The content of phenolic compounds in hops is influenced in particular by the cultivar, the growth location, the field conditions, the climate, which may explain the differences in activities found in the literature [47,48].

Phenolic compounds are known to be an anti-staphylococcal class of metabolites. In our study, all purified hops prenylated chalcones and acylphloroglucinol derivatives showed an antibacterial activity towards selected *S. aureus* strains (Table 3). In the literature, the antibacterial activity of hops is mainly linked to acylphloroglucinol derivatives. This activity is enhanced by the degree of hydrophobicity of the compound [49]. The number and the length of the side chains, because of their interaction with the bacterial cell wall, is stated to influence positively the antibacterial action, so lupulone derivatives are more efficient than humulone derivatives [22,42], which is in accordance with our results. Lupulone is much more active than humulone with respective MICs ranging from 0.6 to 1.2 and 78 to 156 µg/mL towards MRSA strains (Table 3). With the exception of lupulone, we also demonstrated that xanthohumol and desmethylxanthohumol are more active than other bitter acids. Xanthohumol showed MIC ranging from 9.8 to 19.5 µg/mL against selected *S. aureus* strains. In comparison with one of the most studied promising antimicrobial chalcone, licochalcone A, which showed MIC ranging from 2 to 15 µg/mL against Gram-positive bacteria including *S. aureus* [50], the activity of xanthohumol is in the same range. Several studies have focused on the antibacterial potential of xanthohumol which showed various MICs from 2 to 125 µg/mL on *S. aureus*, depending on the strain’s resistance profile and the compound’s purity [14,22]. According to our results, desmethylxanthohumol was slightly less active than xanthohumol, with MICs ranging from 19.5 to 39 µg/mL against *S. aureus* strains (Table 3). The structural difference between the two molecules is the 6′-methoxyl group for xanthohumol replaced by a 6′-phenol group for desmethylxanthohumol (Figure 1). The 6′-phenol substituent is considered as a crucial group in the equilibrium chalcone-flavanone but is not expected to contribute to the activity [51]. According to Ávila et al. [51], the methylation of the 6-phenol group could lead to less active compounds, which is not the case in our study. Desmethylxanthohumol is known for its antioxidant and apoptotic activities [52] but to our knowledge, no antibacterial potential was highlighted. Olivella et al. [53] have demonstrated that chalcones have the most favorable structure for a bacteriostatic action, in comparison with flavanones. In rats, xanthohumol is partially absorbed by intestinal cells and transported in blood. The non-absorbed part of xanthohumol can be transformed into the corresponding flavanone, isoxanthohumol, by the intestinal microbiota [54]. The conversion of isoxanthohumol into 8-prenyl-naringenin occurs only in the colon and not in the stomach and small intestine [55,56]. In this context, we also tested the activity of isoxanthohumol (Figure 4) against the strain MRSA T28.1. This compound showed a MIC equal to 39 µg/mL. It is interesting to note that, even if isoxanthohumol is less active than xanthohumol, its MIC was found to be of similar than desmethylxanthohumol.

The antibacterial potential of xanthohumol, desmethylxanthohumol and lupulone against MRSA is therefore promising. Their MICs are in the same order of magnitude as those obtained for antibacterial phenolic compounds known for their important activity against MRSA strains, with MICs varying between 1.56 and 125 µg/mL [4]. Most of them are known to be antibacterial compounds but they are not used to combat strains resistant to antibiotics. Some hops metabolites also showed antibacterial activities against others strains such as: *Bacillus subtilis*, *Clostridium difficile*, *Enterococcus strains* and *Streptococcus strains* for the Gram-positive bacteria; *Bacteroides fragilis*, *Helicobacter pylori* and *Yersinia enterocolitica* for the Gram-negative bacteria [20].

To overcome bacterial resistance, one of the strategies employed is the use of a combination of drugs, such as antibiotics combined with natural products, which has already shown promising results [57]. The checkerboard is a method to establish the best combination of products increasing the activity [32,58]. The antibacterial action of hops compounds can be enhanced by combining concomitantly xanthohumol with desmethylxanthohumol or with lupulone. The effect is twice as intense because the MIC is divided by two. By contrast, the combination of desmethylxanthohumol with lupulone leads to an antagonist effect (Table 4). The combination of natural products with antibiotics may in some cases have a synergistic effect. Xanthohumol and rifampicin can be 8 times more active when they are combined. Furthermore, for both xanthohumol and lupulone, the MIC for oxacillin drops below the threshold concentration of 2 µg/mL for oxacillin resistance, which means that in the presence of these synergistic compounds the strains will no longer be classified as MRSA, so reverting their resistance. Desmethylxanthohumol has an additive effect with oxacillin but this interaction does not render the strain susceptible to this antibiotic. Desmethylxanthohumol also has a promising interaction with ciprofloxacin and gentamicin. Some authors have previously detected synergies of xanthohumol and lupulone with polymyxin, ciprofloxacin or tobramycin [59]; and of xanthohumol with oxacillin or linezolid [14].

Kill curves demonstrated a great bactericidal action of xanthohumol at the MIC, of desmethylxanthohumol from 2 × MIC, whereas lupulone is slightly bactericidal after 24 h only at 4 × MIC (Figure 5). Comparing the activity of the two chalcones, desmethylxanthohumol showed a lower bactericidal action at the MIC than xanthohumol, which is probably linked to the presence of the 6′-hydroxyl group (Figure 1). Post-antibiotic effect is a part of pharmacodynamic studies, showing that xanthohumol and desmethylxanthohumol cause a significant delay for regrowth (Table 6). It means that the bacterial growth remains inhibited even after the product has been inactivated or metabolized by the body. These data provide an indication of the delay between two applications in a clinical situation. This is the first time that PAE is analyzed for hop compounds. This effect underlines an important reduction time for recovery which means that in vivo models will have to be checked for delay in drug administration.

Xanthohumol, desmethylxanthohumol and lupulone showed an inhibition of the biofilm formation of the *S. aureus* model strain on abiotic surface, with a sub-inhibitory action for desmethylxanthohumol and lupulone (Figure 7). Rozalski et al. studied the anti-adherent potential of a hops extract enriched in xanthohumol, pure xanthohumol and a spent hops extract rich in various common flavonols and flavanols [14]. They demonstrated a potent effect of xanthohumol on the biofilm formation at the MIC with 86.5% of inhibition. In comparison, our results showed an inhibition close to 100% at the MIC for the selected MRSA clinical isolate. In addition, we have also demonstrated that a previous formation of the biofilm does not prevent hops compounds to act on bacteria. In both cases, desmethylxanthohumol and lupulone seem to be more effective than xanthohumol, with an inhibition of the biofilm formation and a biofilm destruction at sub-inhibitory concentrations (Figure 7). Bogdanova et al. [23] also showed an anti-biofilm potential of some hops compounds but lower than that of our study. This result could be related to a lower purity of their products (from 82 to 87% in Reference [23]). This potential has been confirmed on a synthetic bone substitute which is the natural colonized substrate of *S. aureus* T28.1 (Figure 7). Even if the inhibition of the biofilm formation is found somewhat less intense in bone substitute than on inert surface, this potential remains very interesting, in particular for desmethylxanthohumol and lupulone. Moreover, according to our results, the anti-biofilm effect for these two products seems to be independent of the bactericidal effect pointed out with kill curves (Figure 5). To our knowledge, the anti-biofilm effect has never been assessed for hops compounds on bone substitutes. These data confirmed the promising potential of hops compounds to tackle MRSA not only on planktonic cells (MIC, kill curves) but also on biofilms approaching clinical situations.

Diabetic foot infections (DFI) affect one ulcerated foot out of two and in many cases lead to serious complications [60]. About 50% of patients hospitalized for a DFI suffer from an osteomyelitis and the prevalence of MRSA is often associated [61]. Low diffusion in necrotic tissues emphasizes topical antibiotic therapy for the management of mildly to moderately infected diabetic foot ulcers which has shown satisfactory results in some cases, allowing high concentrations of antibiotics at the site of infection without potentially toxic systemic levels [62]. Some medical devices such as beads loaded with antibiotics can bring high concentrations of local antibiotics for a long time in the case of deep wounds [63,64]. In addition, some topical antimicrobial agents, such as impregnated wound dressings with antimicrobials, could be of interest in the prevention or possibly the treatment of mild infections [65]. DFI generates many problems in clinical practice in terms of both diagnosis and therapeutic care mainly due to formation of persistent biofilms. The anti-biofilm action of hops metabolites both on artificial surface and on a synthetic bone substitute could bring out a new perspective to treat infected diabetic foot ulcers, an emerging public health problem. Thus, hop phenolic compounds with their dual action, antibacterial and anti-biofilm, are potential agents in the treatment of infections due to MRSA. Their additive or synergistic action with antibiotics could render treatments more effective and thus could prevent potential systemic toxicity if used in topical application. In this context, we evaluated the antiproliferative activity of the three phenolic compounds on different human cell lines and in particular against the human osteoblasts MG-63 cell line. After 48 h exposure, we showed a toxicity of xanthohumol on the targeted cell line. In the literature, data on its cytotoxicity depend on the cell type used and is very variable. For example, Ho et al. and Yong et al. have determined respectively an IC_50_ of about 75 and 100 µg/mL against a human hepatocellular carcinoma [66] and a lung cell line [67]. These concentrations are higher than the active doses reported in our work. In vivo studies have also confirmed the good safety at approximately 1000 mg of xanthohumol/kg of body weight of mice [68] and up to 180 mg of compound in humans for a short intake [69]. According to our results, desmethylxanthohumol is the less toxic compound on the MG-63 osteoblastic cells. However, considering its moderate activity, bactericidal and anti-biofilm concentrations would be toxic. To our knowledge, there is no comparison data available in the literature. Special attention has to be paid on lupulone. Its very pronounced antibacterial activity makes it non-toxic at the active doses. Moreover, anti-biofilm concentrations (MIC/4 and MIC/2) lead to a cell viability close to 100%. Comparing with the literature, some authors have determined IC_50_ ranging from 3.7 to 4.4 µg/mL on prostate cancer cells, which is close to other results [70] and IC_50_ from 8.3 to 16.6 µg/mL on breast cancer cells [71]. IC_50_ values are always higher than the active doses we have identified. All these data make lupulone a very good candidate for a topical bone application. Further research could be done by combining xanthohumol or desmethylxanthohumol with antibiotics as it would reduce the dose and avoid toxicity.

In addition, we evaluated the antiparasitic activity of the main chalcones and the main acylphloroglucinols of hop against two parasites: *Trypanosoma brucei brucei* (Tbb) and *Leishmania mexicana mexicana* (Lmm). Human African trypanosomiasis and leishmaniasis are indeed two protozoan infections considered as neglected tropical diseases with a strong impact on human health because in particular fatal if untreated [16,17]. Lupulone was the most active compound and humulone the less active. The four compounds tested were more active against Tbb than against Lmm (Table 6). Data on the antiparasitic activities of hops compounds are quite limited and especially concern xanthohumol. This chalcone was active against *Plasmodium falciparum* [24] and against *Leishmania amazonensis* [72] with IC_50_ in the µM range.

## 4. Materials and Methods

### 4.1. Phytochemical Analysis

#### 4.1.1. General Experimental Procedures

For extraction and fractionation, synthesis grade ethanol (EtOH) and dichloromethane (DCM) were furnished by VWR Prolabo^®^ (Fontenay-sous-Bois, France). Water was bi-distilled. All organic solvents for Centrifugal Partition Chromatography (CPC) purification were High Pressure Liquid Chromatography (HPLC) grade except for the n-heptane which was synthesis grade (Carlo Erba Reagents^®^, Val-de-Reuil, France). Ethyl acetate (EtOAc) and methanol (MeOH) were purchased from Fisher Scientific^®^ (Illkirch, France). Water was purified using Millipore Integral 5 (Merck^®^, Trosly-Breuil, France) water purification system with a resistivity of not less than 18 MΩ·cm^−1^. For analyses, acetonitrile (LC-MS grade) was purchased in Carlo Erba Reagents^®^ (Val de Reuil, France), whereas methanol (LC-MS grade) came from Fischer Scientific^®^ (Illkirch, France). The chloroform-d6 (CDCl_3_) and MeOD for Nuclear Magnetic Resonance (NMR) experiments was obtained from Euriso-Top^®^ (Gif-sur-Yvette, France).

Analytical Thin Layer Chromatography (TLC) were performed on pre-coated silica gel 60 F (0.25 mm, Merck^®^, Darmstadt, Germany). Detection was achieved at 254 and 366 nm, then by spraying with the unspecific anisaldehyde sulphuric reagent and heating at 100 °C for 10 min.

Ultra-High Performance Liquid Chromatography (UHPLC) analyses and quantification were carried out using an Acquity UPLC^®^ H-Class Waters^®^ system (Waters, Guyancourt, France) equipped with a diode array detector (DAD) and an Acquity QDa ESI-Quadrupole Mass Spectrometer. The software used was Empower 3. The stationary phase was a Waters^®^ Acquity BEH C18 column (2.1 × 50 mm, 1.7 μm) connected to a 0.2 µm in-line filter. Preparative HPLC was performed using a Shimadzu^®^ HPLC system equipped with a LC-20AP binary high-pressure pump, a CBM-20A controller and a SPD-M20A diode array detector. The software used was LabSolution. The stationary phase was a VisionHT HL C18 (5 μm, 250 × 22 mm) column (Grace^®^, France).

CPC was performed using an Armen instrument 250 mL rotor (SCPC-250-L) provided by Gilson^®^ (Saint-Avé, France). CPC analyses were monitored using Shimadzu^®^ pump and detector.

Nuclear Magnetic Resonance (NMR) spectra were recorded on a Bruker^®^ DPX-500 spectrometer. High Resolution Mass Spectrometry (HR-MS) analyses were carried out using a Thermo Fisher Scientific^®^ Exactive Orbitrap Mass Spectrometer equipped with an electrospray ion source.

#### 4.1.2. Plant Extract Preparation and Fractionation

Female hop plants (*Humulus lupulus* L., cultivar ‘Nugget’) were harvested at maturity stage at the Beck farm (Bailleul, Northern France), at the time of harvesting of hop by producers in September. A voucher specimen was kept at the Faculty of pharmacy in Lille (laboratory of pharmacognosy) under reference NugBeck2015. After drying for 10 days at room temperature, protected from light, rhizomes, stems, leaves and cones were powderized separately with a blender. Crude hydro-alcoholic extracts of each part were obtained after an ethanol/water (9:1; *v*/*v*; 15 mL/g) mixture-based extraction with three successive macerations of four hours and one overnight, stirring in the dark. The percentage yields (PY) on a dry weight basis of each crude extract were: 35.5% (cones), 20.3% (leaves), 21.1% (rhizomes) and 17.2% (stems). The crude extract of female cones was fractionated by a liquid-liquid separation using dichloromethane (DCM) and water to obtain two sub-extracts. The corresponding sub-extracts were obtained with percentages yields of 52% and 48% respectively

#### 4.1.3. Purification of Phenolic Compounds

Xanthohumol, desmethylxanthohumol, humulone, cohumulone, lupulone, colupulone were purified from the DCM sub-extract of cones in several steps. A first fractionation was performed by CPC. Using the Arizona solvent system P: n-heptane/EtOAc/MeOH/water (6:5:6:5; *v*/*v*), the rotor was entirely filled at 30 mL/min with the aqueous stationary phase in the ascending mode with rotation (500 rpm). Then, the rotation speed was increased from 500 to 1600 rpm. The organic mobile phase was pumped into the column in ascending mode at a flow rate of 8 mL/min. DCM sub-extract (2 g), initially dissolved in 10 mL of the organic/aqueous phase mixture (1:1), was filtered with a Millipore (0.45 μm) syringe filter and was injected immediately after the displacement of stationary phase (approximatively 80 mL). Fractions of 8 mL were collected every min. The CPC was run in ascending mode for 60 min and then switched to extrusion mode (recovery of the stationary phase) for 10 additional minutes at 30 mL/min with the same rotor speed (1600 rpm). The content of the outgoing organic phase was monitored by online UV absorbance measurement at 254 nm and 370 nm. All the fractions were checked by TLC and developed with toluene/ethyl acetate/formic acid (73:18:9; *v*/*v*) in order to regroup 5 sub-fractions (MC1 to MC5) from ascendant mode and 3 sub-fractions (MC6 to MC8) from extrusion mode. This CPC method allowed us to purify, in one step, xanthohumol (Figure 1), with 98% purity from MC4. The other compounds were purified from other sub-fractions using preparative HPLC. The mobile phase was composed of water (solvent A) and acetonitrile (solvent B). The following proportions of solvent B were: 10–75% (0–5 min), 75% (5–30 min), 75–100% (30–35 min) and 100% (35–45 min) at 12 mL/min. Injections with 500 µL of a 60 mg/mL fraction solution in methanol were performed. This process allowed us to purify several acylphloroglucinol derivatives (α- and β-acids) from sub-fractions MC1 and MC2, as well as another chalcone, desmethylxanthohumol, from the sub-fraction MC7, with a purity greater than 95% (Figure 1). Throughout the process, we protected the samples of the light as much as possible.

#### 4.1.4. UHPLC-UV-MS Analyses

The crude hydro-ethanolic extracts of hop parts (cones, leaves, stems and rhizomes) were analyzed by UHPLC-UV-MS. The mobile phase consisted of 0.1% formic acid in water and of 0.1% formic acid in acetonitrile. The gradient of acetonitrile was: 50% (0–1 min), 50–75% (1–3 min), 75% (3–5 min), 75–100% (5–7 min) and 100% (7–9,5 min) at 0.3 mL/min. Column temperature was set at 30 °C. Solutions of crude extracts were prepared in MeOH at 100 µg/mL for cones and 1 mg/mL for the other parts. Injection volume was 2 µL. The main chalcones and acylphloroglucinols were identified on the basis of the retention time of the purified standards and their mass spectra.

The ionization was performed in negative mode. Cone voltage was set at 10 V. Probe temperature was 600 °C. Capillary voltage was 0.8 kV. The MS-Scan mode was used from 100 to 1000 Da.

#### 4.1.5. Quantification Using UHPLC-UV

The most abundant prenylated chalcones and acylphloroglucinol derivatives were quantified in hop crude hydro-ethanolic extracts (cones, leaves, stems and rhizomes), according to the international guidelines for analytical techniques for quality control of pharmaceuticals [73]. Quantification was performed in UV at 370 nm for chalcones and 330 nm for acylphloroglucinols. *Co*- and *ad*-acids were quantified from the respective calibration curves of the *n*-acids (humulone for alpha acids and lupulone for beta acids), using molecular weight ratio. Desmethylxanthohumol was quantified from xanthohumol calibration curve, using molecular weight ratio. Solutions of crude extracts were prepared in MeOH at 100 µg/mL for cones and 1 mg/mL for the other parts. Sample solutions were prepared in triplicate the same day. Aliquots of each solution (2 µL) were injected in triplicate.

Stock solutions of xanthohumol, humulone and lupulone, previously purified, were prepared at the concentration of 1 mg/mL in MeOH for quantification, then stored at −20 °C until use. Fifteen working solutions (100 µg/mL to 2.5 ng/mL) were daily prepared by dilutions. Three mixed solutions containing the three analytes were prepared at the concentrations of 100, 50 and 25 µg/mL from stock solutions. Then, lower concentrations were obtained from these intermediate solutions by successive dilutions in MeOH. Calibration curves were designed to cover the expected range of concentrations in samples after preliminary injection of crude extracts solutions. Nine, ten and twelve concentration levels were respectively used for calibration curves of lupulone, xanthohumol and humulone. They were built by plotting peak area (*y*) as a function of the nominal concentration for each calibration level (*x*) and then fitted by weighted (1/*x*) least square linear regression. Linearity and precision of the method, as well as the limit of detection (LOD) and the limit of quantification (LOQ) were reported in Table S1 (Supplementary Material). LOD was defined as the lowest concentration with a S/N > 3. LOQ was defined as the lowest concentration with a deviation <20% on back calculation. Intra and inter-day precisions were evaluated on cone sample solutions. They were prepared on three different days, in triplicate each day (*n* = 3, *k* = 3). 2 µL of each solution was injected 3 times.

#### 4.1.6. Structural Elucidation

Structures of purified compounds were determined using NMR and HR-MS. Mono- (^1^H and ^13^C) and bi-dimensional (COSY, HSQC, HMBC) spectra were carried out for each compound. Prenylated chalcones were solubilized in deuterated methanol (MeOD) whereas acylphloroglucinol derivatives were solubilized in deuterated chloroform (CDCl_3_). HR-MS analyses were carried out in positive mode with a range of *m*/*z* 100–1000 amu. Products were solubilized in methanol with a drop of DCM for the acylphloroglucinols.

### 4.2. Antimicrobial Bioassays

#### 4.2.1. Antibacterial Screening of Extracts and Sub-Extracts Using Agar Dilution Method

Clinical bacterial isolates from human samples collected in Lille (France) and some collection strains were used. The first screening step on pathogenic bacteria was carried out with crude hydro-alcoholic extracts of different hop parts (Table 1). The second screening was performed using sub-extracts of cones on Gram-positive bacteria, including methicillin-sensitive *Staphylococcus aureus* (MSSA) and methicillin-resistant *Staphylococcus aureus* (MSSA) strains (Table 2). These tests were carried out on Petri dishes, Mueller-Hinton Agar (MHA) (Oxoid, UK) was mixed with the plant extract solution in MeOH at 5% (solvent control: 5% MeOH). Final extract concentrations in Petri dishes ranged from 1250 to 4.9 µg/mL. A multi-headed inoculator allowed spotting bacterial strains at 10^5^ CFU/mL in cysteinated Ringer (CR) solution (Merck^®^, France). Minimal Inhibitory Concentrations (MICs) were visually determined after 24 h of incubation at 37 °C.

#### 4.2.2. Antibacterial Susceptibility of Compounds Using Broth Microdilution Method

For bioassays, xanthohumol, desmethylxanthohumol, humulone, cohumulone, lupulone and colupulone were used had a minimum purity of 95% (HPLC-UV). The protocol employed was inspired by Abedini et al. [74] with some modifications. Products were solubilized in DMSO and serially diluted two-fold in MH medium. A bacterial suspension at 10^5^ CFU/mL was added to obtain a final volume of 200 µL. Final phenolic product concentrations in wells ranged from 625 to 2.4 µg/mL (exception for lupulone for which dilutions were continued until 0.3 µg/mL). The susceptibility of the strain to DMSO has previously been assessed, the DMSO concentration in wells taken into account did not exceed 5% (concentration without effect on growth). Plates were incubated overnight with stirring (60 rpm) at 37 °C. The bacterial growth was indicated visually and by a developer of enzymatic activity (iodonitrotetrazolium chloride—INT, AppliChem, Germany) which reveals bacterial growth by a purple color after 15 min heating at 55 °C.

For the following experiments, the MRSA strain T28.1, isolated from a pathological sample of osteitis, was used. It was previously characterized by DNA biochips, which allowed highlighting some β-lactamases and the SCC-mec genes confirming the methicillin resistance [30]. Moreover, genes for enzymes and proteins involved in the capsule biosynthesis (capH5, capJ5 and capK5) and several intracellular adhesion proteins implicated in biofilm formation (icaA, icaC and icaD) were present.

The commercial product, isoxanthohumol, was also tested against *S. aureus* T28.1 (purity superior to 95%, Phytolab^®^, Germany).

#### 4.2.3. Synergies with Selected Antibiotics (Checkerboard Method)

Checkerboard method was used to assess the potential co-action of xanthohumol, desmethylxanthohumol and lupulone between them or with some antibiotics [32,58]. Antibiotics were previously selected on the basis of a first screening using E-tests (BioMérieux^®^, France). They covered several classes and are currently used to treat either *S. aureus* infections or bone infections: oxacillin (purity 95%, Acros Organics, Belgium), ciprofloxacin, gentamicin and rifampicin (purity 99.9%, 65.7% and 99.2% respectively, PanReac AppliChem^®^, Germany). Each test was performed on a 96-well microplate using an 8-by-8 well configuration. Concentration of hops phenolic compounds and antibiotics tested ranged from MIC/4 to 4xMIC. Wells were filled with 100 µL of MH medium, 10 µL of each compound (DMSO for products and water for antibiotics) and 80 µL of a 10^5^ CFU/mL bacterial suspension with appropriate solvent controls. Final concentration in DMSO was 5%, which did not affect the bacterial growth. The MIC of each product alone was checked at each test. Microplates were incubated overnight at 60 rpm and 37 °C. The bacterial growth was visually assessed and confirmed by revealing bacterial enzymatic activity by adding INT.

After visual analysis, the combination with the highest activity was determined by the calculation of the fractional inhibitory concentration (FIC) index [32], interpreted as synergistic (FIC < 0.5), additive (0.5 ≤ FIC ≤ 1), indifferent (1 < FIC ≤ 4) or antagonist (FIC > 4).

$$\mathrm{FIC}\;\mathrm{index}=\frac{\mathrm{MIC}\;A\;\mathrm{with}\;B}{\mathrm{MIC}\;A\;\mathrm{alone}}+\;\frac{\mathrm{MIC}\;B\;\mathrm{with}\;A}{\mathrm{MIC}\;B\;\mathrm{alone}}$$

#### 4.2.4. Kill Curves

This experiment allowed following in time the effect of the product on a growing bacterial population, highlighting the bactericidal or bacteriostatic effect [75]. Stock solutions of xanthohumol, desmethylxanthohumol and lupulone were prepared in DMSO 20 times more concentrated than the desired final concentrations (MIC/4 to 4xMIC). An aliquot of 0.5 mL of purified product in DMSO was added to 8.5 mL of brain heart infusion (BHI) medium (Oxoid, UK). 1 mL of bacterial suspension at 10^5^ CFU/mL was then introduced to the culture tube. A 5% DMSO control served as a negative control and was performed at each test. Culture tubes were incubated at 37 °C for 24 h. Counts were made every 2 h until 8 h and at 24 h by plating aliquots of serial tenfold dilutions on MHA. The determined bacterial concentrations were then converted into log(CFU/mL) and were expressed as a function of time. The detection threshold of this method was 10 CFU/mL and 1 log(CFU/mL) on graphics.

#### 4.2.5. Post-Antibiotic Effect (PAE)

This method allows quantifying the delayed regrowth of a bacterial population following exposure to an active compound [76]. Stock solutions of xanthohumol, desmethylxanthohumol and lupulone were prepared in DMSO 20 times more concentrated than the desired final concentrations (MIC/4 to 4 × MIC). A 2 h pre-treatment with purified prenylated compounds was performed using 8.5 mL of BHI medium, 0.5 mL of purified product in DMSO and 1 mL of a bacterial suspension at 10^5^ CFU/mL, incubated at 37 °C. Final concentrations in DMSO did not exceed 5%. The hop compound was then inactivated by a 1000-fold dilution. After inactivation, a growth curve was performed with counts at 30 min and every 2 h. The growth lag was quantified by comparison with the control, using the following formula:

PAE=T−C

T is the time needed for the bacterial population to grow by 1 log(CFU/mL) at the given concentration, C is the corresponding time for the control (Figure 5)

#### 4.2.6. Anti-Biofilm Tests

These experiments were inspired and adapted from Liu et al. [77]. They were performed using a 96-well microplate with a flat bottom.

##### On Artificial Surface

First, the effect of xanthohumol, desmethylxanthohumol and lupulone was assessed on the biofilm formation: 180 µL of BHI medium containing glucose at 10 mg/mL was added to each well of a 96-well microplate with 10 µL of the product previously solubilized in DMSO and 10 µL of a 10^5^ CFU/mL bacterial suspension. The maximum concentration in DMSO was 5%. Final concentrations of purified products were from MIC/4 to 4 × MIC. After 24 h incubation at 37 °C, wells were voided and washed 3 times with phosphate buffered saline solution (PBS, Sigma-Aldrich, Saint-Quentin Fallavier, France). Plates were then dried and 150 µL of crystal violet solution at 20 mg/mL in MeOH (Sigma-Aldrich^®^, Saint-Quentin Fallavier, France) were added for 15 min. After removing crystal violet, 150 µL of EtOH were added to solubilize crystal violet present in adherent bacteria. The optical density was read at 570 nm.

The effect was also evaluated after the biofilm formation in order to evaluate if the hop phenolic compounds are able to destroy it. A first culture of 24 h at 37 °C with 190 µL of BHI medium and 10 µL of a 10^5^ CFU/mL bacterial suspension allowed producing the biofilm. Wells were voided and washed with PBS. The biofilm was then treated with xanthohumol, desmethylxanthohumol or lupulone diluted at 5% in the BHI medium to obtain a final volume of 200 µL. Plates were incubated for 24 h at 37 °C, then voided and colored with crystal violet as above.

##### On Synthetic Bone Substitute

β-Tricalcium phosphate discs (Cerasorb^®^, Curasan, Germany) were used as bone substitute. Non-glucose enriched BHI medium was used here, because the bacterial glucose metabolism causes an acidification of the culture medium which leads to the disintegration of the discs. Discs were introduced in a 24-well plate: 1.8 mL of BHI media were added with 0.1 mL of product in DMSO (5%) and 0.1 mL of a 10^5^ CFU/mL bacterial suspension. Final concentrations of hop phenolic compounds were from MIC/4 to 4 × MIC. After 24 h at 37 °C, counts were performed in two steps. First, planktonic bacteria were counted from 100 µL of the culture supernatant by plating tenfold dilutions on MHA. Then, bacteria adhering to the discs were counted: discs were removed and washed with a CR solution, then, 10 mL of CR were added to each disk and treated 1 min by sonication (35 kHz) and 30 s by vortex. The obtained suspension was enumerated as previously. The detection threshold was also 1 log(CFU/mL).

#### 4.2.7. Antiparasitic Activity of Hops Using Broth Microdilution Method

Tbb were cultivated in HMI9 medium containing 10% heat-inactivated foetal bovine serum (FBS) (Sigma-Aldrich), 150 mM L-cysteine and 20 mM beta-mercaptoethanol at 37 °C (CO_2_ 5%). Lmm (MHOM/BZ/84/BEL46) were cultivated in SDM-79 medium (Gibco) supplemented with 15% heat-inactivated FBS (Sigma-Aldrich) and 5 mg/L hemin at 28 °C (CO_2_ 5%). Antiparasitic bioassays were performed as described by Bero et al. [17]. Suramin (SUR) and pentamidine (PEN) were used as positive controls respectively. The hops compounds (xanthohumol, desmethylxanthohumol, humulone, lupulone) concentration that inhibits 50% of the cell viability (IC_50_) was determined using GraphPad Prism, version 5.01 (GraphPad Software, San Diego, CA, USA).

### 4.3. Antiproliferative Effect of Purified Compounds on Human Cell Lines

Several human cell lines were used for the cytotoxic bioassays: non-cancer lung fibroblasts (WI-38), hepatocellular carcinoma (Hep-G2), osteosarcoma (MG-63) and the mouse monocyte macrophage J774. Cells were seeded into wells of a 96-well microplate in a Gibco™ Dulbecco’s modified eagles medium (DMEM), except for MG-63 which were cultivated in a minimum essential medium (MEM) (ThermoFisher Scientific, Illkirch-Graffenstaden, France). Both media were enriched with 10% FBS (ThermoFisher Scientific, France) and some antibiotics (mixture penicillin/streptomycin 100 UI/mL, Sigma Aldrich). After one or two days at 37 °C (5% of CO_2_), wells were emptied by suction. Cells were then treated with hops compounds (xanthohumol, desmethylxanthohumol, humulone, lupulone) in DMSO at 0.2% in the culture medium, to obtain a final volume of 100 µL in wells (negative control: 0.2% DMSO). Camptothecin was used as a positive control. After 48 to 72 h exposure, culture medium was replaced by 10% of Alamar blue or 3-(4,5-dimethylthiazol-2-yl)-2,5-diphenyltetrazolium bromide (MTT) in the medium (ThermoFisher Scientific^®^, Illkirch, France) and plates were incubated the time necessary to the reaction. Results were measured respectively by fluorescence (excitation 530 nm and emission 590 nm) or by optical density at 550 nm. The IC_50_ of each product was determined using GraphPad Prism (version 5.01).

### 4.4. Statistical Analyses

Statistical analyses were carried out using the software R, version 3.4.1 (The R Foundation for Statistical Computing, Vienna, Austria). Each experiment was performed in independent triplicates. For each distribution, the normality of the residues was assessed using Shapiro test. If the normality was accepted, an ANOVA and the Tukey HSD test were performed. If the normality was refused, Kruskal Wallis and Dunn’s tests were used at a significance level of *p* = 0.05.

## 5. Conclusions

Xanthohumol, desmethylxanthohumol and lupulone from hops, with their dual antibacterial and anti-biofilm actions, are potential agents in the treatment of infections due to MRSA. Their additive or synergistic actions with antibiotics could render treatments more effective and thus could prevent toxicity at systemic level if used in topical application. Their chemical structures differ from current anti-staphylococcal agents and enable us to assume that they act on a different target site of action in *S. aureus*. The exact identification of this target is a future challenge. Moreover, for the first-time, activity of hops phenolic compounds was highlighted on the Tbb and Lmm parasites.

## Figures and Tables

**Table 1:** MIC (in µg/mL) of hop hydro-ethanolic crude extracts (Co: cones, Le: leaves, St: stems, Rh: rhizomes) against some human pathogenic bacteria with their corresponding antibiotic susceptibility (S: susceptible I: intermediate, R: resistant).

Bacterial Strains and Yeasts	MIC (µg/mL)				Antibiotic and Antifungal Susceptibility		
	Co	Le	St	Rh	GEN	VAN	AMX
**Gram-positive**							
*Corynebacterium* T25-17	39	NA	NA	NA	S	S	S
*Enterococcus faecalis* C159-6	39	NA	NA	NA	R	S	R
*Enterococcus sp.* 8153	156	NA	NA	NA	R	S	S
*Mycobacterium smegmatis* 5003	39	NA	NA	NA	S	S	S
*Staphylococcus aureus* 8146	39	NA	NA	NA	S	S	I
*Staphylococcus aureus* 8147	39	NA	NA	NA	S	S	S
*Staphylococcus epidermidis* 5001	39	NA	NA	NA	S	S	S
*Staphylococcus epidermidis* 10282	98	NA	NA	NA	S	S	S
*Staphylococcus lugdunensis* T26A3	156	NA	NA	625	S	S	S
*Staphylococcus warneri* T12A12	39	NA	NA	625	S	R	S
*Streptococcus agalactiae* T25-7	39	NA	NA	NA	R	S	S
*Streptococcus agalactiae* T53C2	78	NA	NA	NA	S	S	S
*Streptococcus dysgalactiae* T46C14	39	NA	NA	NA	S	S	S
**Gram-negative**							
*Acinetobacter baumannii* 9010	NA	NA	NA	NA	S	R	R
*Acinetobacter baumannii* 9011	NA	NA	NA	625	R	R	R
*Citrobacter freundii* 11041	NA	NA	NA	NA	S	R	S
*Citrobacter freundii* 11042	NA	NA	NA	NA	S	R	R
*Enterobacter cloacae* 11050	NA	NA	NA	NA	S	R	R
*Enterobacter cloacae* 11051	NA	NA	NA	NA	R	R	R
*Enterobacter cloacae* 11053	NA	NA	NA	NA	S	R	R
*Escherichia coli* 8138	NA	NA	NA	NA	S	R	R
*Escherichia coli* 8157	NA	NA	NA	NA	S	R	R
*Escherichia coli* ATCC 25922	NA	NA	NA	NA	S	R	I
*Klebsiella pneumoniae* 11016	NA	NA	NA	NA	S	R	R
*Klebsiella pneumoniae* 11017	NA	NA	NA	NA	S	R	R
*Proteus mirabilis* 11060	NA	NA	NA	NA	S	R	R
*Providencia stuartii* 11038	NA	NA	NA	NA	S	R	S
*Pseudomonas aeruginosa* 8131	NA	NA	NA	625	S	R	R
*Pseudomonas aeruginosa* ATCC 27583	NA	NA	NA	625	R	R	R
*Salmonella sp.* 11033	NA	NA	NA	NA	S	R	R
*Serratia marcescens* 11056	NA	NA	NA	NA	S	R	R
*Serratia marcescens* 11057	NA	NA	NA	NA	R	R	R
*Stenotrophomonas maltophilia*	NA	NA	NA	625	S	R	R
**Yeasts**					**AMB**	**FLC**	**VRC**
*Candida albicans* 13203	156	625	NA	NA	S	R	R
*Candida albicans* ATCC 10231	<39	NA	NA	NA	S	S	S

Gentamicin (GEN) S ≤ 4, R > 8; Vancomycin (VAN) S ≤ 4, R > 16; Amoxicillin (AMX) S ≤ 4, R > 16; Amphotericin B (AMB) S ≤ 1, R > 1; Fluconazole (FLC) S ≤ 2, R > 4; Voriconazole (VRC) S ≤ 0.12, R > 0.12. NA means not active, MIC ≥ 1250 µg/mL.

**Table 2:** MIC (in µg/mL) of the crude extract and the dichloromethane (DCM) sub-extract of cones against Gram-positive strains with their corresponding antibiotic susceptibility (S: susceptible, I: intermediate, R: resistant).

Gram-Positive Strains	MIC (µg/mL)		Antibiotic Susceptibility		
	Crude Extract	DCM	GEN	VAN	AMX
*Corynebacterium* T25-17	39	19.5	S	S	S
*Enterococcus faecalis* C159-6	156	78	R	R	R
*Enterococcus faecalis* T26-B7	313	156	R	S	S
*Enterococcus faecalis* T34-2	313	156	S	S	S
*Enterococcus faecalis* T37A4	156	156	R	S	S
*Enterococcus faecalis* T39-C11	156	156	S	S	S
*Staphylococcus aureus* 8146	19.5	9.8	S	S	S
*Staphylococcus aureus* 8147	19.5	9.8	S	S	S
*Staphylococcus aureus* CIP 224	39	19.5	S	S	S
*Staphylococcus aureus* T1.1	39	19.5	S	S	S
*Staphylococcus aureus* T25.10	39	19.5	S	S	R
*Staphylococcus aureus* T25.3	39	19.5	S	S	R
*Staphylococcus aureus* T25.9	39	19.5	S	S	R
*Staphylococcus aureus* T26A4	39	19.5	S	S	S
*Staphylococcus aureus* T28.1	19.5	9.8	S	S	R
*Staphylococcus aureus* T36B1	39	19.5	S	S	R
*Staphylococcus aureus* T47A12	39	19.5	S	S	R
*Staphylococcus aureus* T6.7	39	9.8	S	S	R
*Staphylococcus epidermidis* 5001	39	19.5	S	S	S
*Staphylococcus warneri* T12A12	78	19.5	S	S	S
*Staphylococcus lugdunensis* T26A3	39	19.5	S	S	S
*Staphylococcus pettenkoferi* T3.3	78	39	S	S	S
*Staphylococcus saprophyticus* 08237	39	9.8	S	S	S
*Streptococcus agalactiae* 13225	39	19.5	S	S	S
*Streptococcus agalactiae* 13226	39	19.5	S	S	S
*Streptococcus agalactiae* T25.7	39	19.5	I	S	S
*Streptococcus agalactiae* T38.2	39	19.5	S	S	S
*Streptococcus agalactiae* T40A2	39	19.5	S	S	S

Gentamicin (GEN) S ≤ 4, R > 8; Vancomycin (VAN) S ≤ 4, R > 16; Amoxicillin (AMX) S ≤ 4, R > 16. NA means not active, MIC ≥ 1250 µg/mL.

**Table 3:** MIC of hop chalcones and acylphloroglucinols against selected MRSA (T28.1 and T25.10) and MSSA (T26A4 and 08143) strains.

Bacteria MIC in µg/Ml (µM)	Chalcones		Acylphloroglucinols				OXA
	XN	DMX	Cohumulone	Humulone	Colupulone	Lupulone	
*S. aureus* T28.1	9.8 (27.7)	39 (114.7)	156 (448.3)	78 (215.5)	39 (97.5)	1.2 (2.9)	R
*S. aureus* T25.10	9.8 (27.7)	19.5 (57.3)	313 (899.4)	156 (430.9)	78 (195)	0.6 (1.45)	R
*S. aureus* T26A4	9.8 (27.7)	39 (114.7)	313 (899.4)	156 (430.9)	39 (97.5)	0.6 (1.45)	S
*S. aureus* 08143	19.5 (55)	39 (114.7)	313 (899.4)	156 (430.9)	78 (195)	1.2 (2.9)	S

XN: xanthohumol, DMX: desmethylxanthohumol. Positive control was oxacillin (OXA, S ≤ 2 µg/mL, R ≥ 4 µg/mL [31]).

**Table 4:** Effect of the combination of hops compounds between them and with selected antibiotics.

Association	FIC Index	Effect
XN-DMX	0.74–1	Additive
XN-Lupulone	0.75	Additive
DMX-Lupulone	5	Antagonist
CIP-XN	0.49–1	Synergistic to additive
CIP-DMX	0.38–1.5	Synergistic to indifferent
CIP-Lupulone	0.63–1	Additive
GEN-XN	0.14–1	Synergistic to additive
GEN-DMX	0.03–0.28	Synergistic
GEN-Lupulone	9	Antagonist
OXA-XN	0.28–0.75	Synergistic to additive
OXA-DMX	0.5–0.76	Additive
OXA-Lupulone	0.19–1.25	Synergistic to indifferent
RIF-XN	0.25–0.75	Synergistic to additive
RIF-DMX	1–5	Indifferent to antagonist
RIF-Lupulone	2.2–6	Indifferent to antagonist

Association can be synergistic (FIC < 0.5), additive (0.5 ≤ FIC ≤ 1), indifferent (1 < FIC ≤ 4) or antagonist (FIC > 4). Ranges result from 3 independent experiments. XN: xanthohumol, DMX: desmethylxanthohumol, CIP: ciprofloxacin, GEN: gentamicin, OXA: oxacillin, RIF: rifampicin.

**Table 5:** Post-antibiotic effect on *S. aureus* T28.1 for each hops selected compound after 2 h exposure. Values are the maximum delayed growth retardation obtained after 3 independent experiments.

Maximum Growth Retardation (h)				
	MIC/2	MIC	2 × MIC	4 × MIC
Xanthohumol	1.34	2.23	2.05	3.29
Desmethylxanthohumol	2.10	2.32	2.29	2.34
Lupulone	0.26	0.53	0.54	0.47

**Table 6:** IC_50_ of hops chalcones and acylphloroglucinols against some *Trypanosoma brucei brucei* and *Leishmania mexicana mexicana* strains.

Parasites IC_50_ in µg/mL (µM)	XN	DMX	Humulone	Lupulone	SUR	PEN
*Tbb*	2.4 ± 0.2 (6.8)	7.7 ± 0.6 (22.6)	10.9 ± 0.8 (30.1)	0.9 ± 0.0 (2.2)	0.05 (0.038)	-
*Lmm*	6.1 ± 3.1 (17.2)	26.2 ± 1.8 (77)	28.8 ± 1.5 (77.9)	4.7 ± 0.1 (11.3)	-	0.07 (0.21)

XN: xanthohumol, DMX: desmethylxanthohumol. Positive controls were suramin (SUR) and pentamidine (PEN). ND: Not determined.

**Table 7:** Cytotoxic activities of hops extracts and isolated compounds (chalcones and acylphloroglucinols) against WI-38, J774, Hep-G2 and MG-63 cell lines.

Cell Lines IC_50_ ± SD in µg/mL (µM)	Hydro-Alcoholic Crude Extract	MC Sub-Extract	XN	DMX	Humulone	Lupulone	CAMP
WI-38	7.6 ± 0.1	5.1 ± 1.0	6.9 ± 0.5 (19.5)	60.7 ± 2.5 (178.5)	10.5 ± 2.3 (29)	1.1 ± 0.0 (2.6)	0.06 ± 0.0 (0.17)
J774	19.7 ± 2.8	11.4 ± 2.1	3.4 ± 0.5 (9.6)	9.7 ± 1.0 (28.5)	11.5 ± 0.3 (31.7)	1.5 ± 0.1 (3.6)	0.01 ± 0.0 (0.03)
Hep-G2	6.8 ± 2.5	6.5 ± 2.1	2.5 ± 0.8 (7.1)	22.4 ± 2.9 (65.9)	ND	1.2 ± 0.5 (2.9)	0.4 ± 0.2 (1.15)
MG-63	31.4 ± 8.1	21.1 ± 0.4	10.4 ± 2.6 (29.4)	39.5 ± 3.3 (116.2)	ND	4.3 ± 0.4 (10.4)	4.4 ± 1.5 (12.6)

XN: xanthohumol, DMX: desmethylxanthohumol. Positive control was camptothecin (CAMP). ND: Not determined.

## Supplementary Materials

The following are available online, Table S1. Linearity and sensitivity of the quantification method by UPLC-UV for xanthohumol, humulone and lupulone, Table S2. ^1^H and ^13^C NMR data for xanthohumol and desmethylxanthohumol in MeOD^a^, Table S3. ^1^H and ^13^C NMR data for humulone and cohumulone in CDCl3^a^, Table S4. ^1^H and ^13^C NMR data for lupulone and colupulone in CDCl3^a^, Figure S1. Chromatograms at 370 nm of the crude hydro-ethanolic extract of cones and purified chalcones (desmethylxanthohumol and xanthohumol), Figure S2. Chromatograms at 330 nm of the crude hydro-ethanolic extract of cones and purified acylphloroglucinol derivatives (cohumulone, humulone, colupulone and lupulone), Figure S3. Total ion chromatogram of the crude hydro-ethanolic extract of cones in negative mode and selected ion recording of purified chalcones (desmethylxanthohumol and xanthohumol) and acylphloroglucinol derivatives (cohumulone, humulone, colupulone and lupulone).
