# Supplementary material for: Phenolic Compounds from Humulus lupulus as Natural Antimicrobial Products: New Weapons in the Fight against Methicillin Resistant Staphylococcus aureus, Leishmania mexicana and Trypanosoma brucei Strains
Source: Molecules. 2019 Mar 14;24(6):1024. doi: 10.3390/molecules24061024 (PMC6472001; doi:10.3390/molecules24061024)

Supplementary material

Table S1. Linearity and sensitivity of the quantification method by UPLC-UV for xanthohumol, humulone and lupulone

**Products**	**LOD (ng/mL)**	**LOQ (ng/mL)**	**Linearity range**	**Slope (a)**	**Intercept (b)**	R^2
Xanthohumol (370 nm)	2.5	10	10 $ng.mL^{-1}$-10 $\mu g.mL^{-1}$	41129.8	159.4	0,997
Humulone (330 nm)	10	25	25 $ng.mL^{-1}$-100 $\mu g.mL^{-1}$	7319.2	41.4	0,998
Lupulone (330 nm)	10	100	25 $ng.mL^{-1}$-10 $\mu g.mL^{-1}$	9747.2	46.8	0,998

Table S2. ^{1}H and ^{13}C NMR data for xanthohumol and desmethylxanthohumol in MeOD[a]

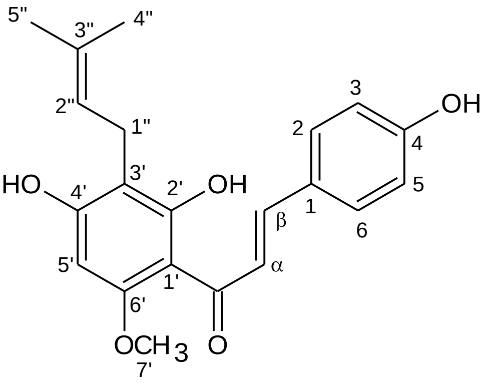

Xanthohumol

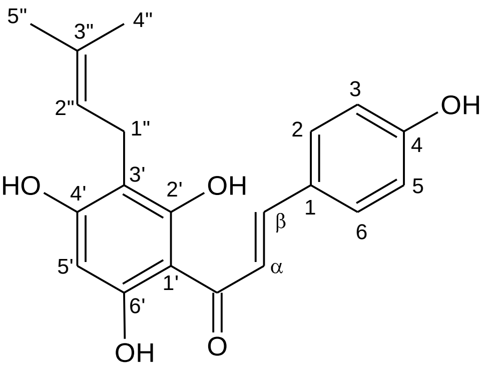

Desmethylxanthohumol

	Chalcones			
	XN		DMX	
Position	δ ^{1}H	δ ^{13}C	δ ^{1}H	δ ^{13}C
1		127		127.2
2	7.50 (*d*, *J* = 8.6 Hz)	129.9	7.48 (*d*, *J* = 8.7 Hz)	129.8
3	6.82 (*d*, *J* = 8.6Hz)	114.8	6.81 (*d*, *J* = 8.7 Hz)	115.4
4		159.5		159.5
5	6.82 (*d*, *J* = 8.6Hz)	114.8	6.81 (*d*, *J* = 8.7 Hz)	115.4
6	7.50 (*d*, *J* = 8.6Hz)	129.9	7.48 (*d*, *J* = 8.7 Hz)	129.8
1’		104.2		104.5
2’		161.3		164.2
3’		107.9		106.9
4’		162.2		162.4
5’	6.02	89.3	5.93	93.8
6’		160.3		159.7
OCH_3	3.90 (*s*)	54.4	-	-
1''	3.25 (*d*, *J* = 7.3 Hz)	20.5	3.20 (*d*, *J* = 7.3 Hz)	20.8
2''	5.20 (*t*, *J* = 7.3 Hz)	122.8	5.19 (*t*, *J* = 7.3Hz)	123.2
3''		129.9		129.7
4''	1.65 (*s*)	24.3	1.65 (*s*)	24.6
5''	1.76 (*s*)	16.1	1.75 (*s*)	16.4
α	7.80 (*d*, *J* = 15.4Hz)	124	8.07 (*d*, *J* = 15.64 Hz)	124.6
β	7.67 (*d*, *J* = 15.4Hz)	142	7.68 (*d*, *J* = 15.64 Hz)	141.7
CO		192.5		192.8

Assignments were also established by COSY, HSQC-DEPT, HMBC

[a] 500 MHz, chemical shifts in ppm relative to TMS, ,^3J in Hz; *s*: singulet, *d*: doublet, *t*: triplet

Table S3. ^{1}H and ^{13}C NMR data for humulone and cohumulone in $CDCl_3$[a]

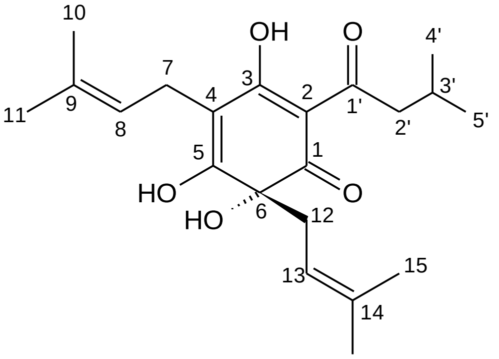

Humulone

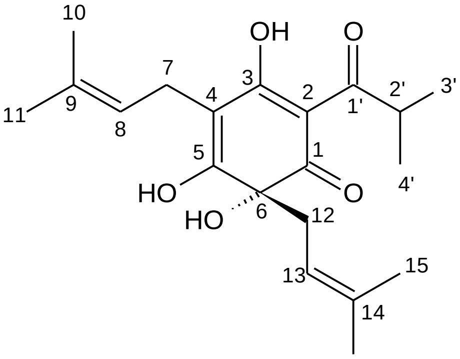

Cohumulone

	α-acids			
	**Humulone**		**Cohumulone**	
Position	δ ^{1}H	δ ^{13}C	δ ^{1}H	δ ^{13}C
1		195.1		195.2
2		109.3		109.9
3		191.0		191.1
4		109.4		109.1
5		167.7		167.7
6		78.8		79.1
7	3.03 (*dd*, *J* = 7.4; 14.2 Hz)<br>3.10 (*dd*, *J* = 7.4 ; 14.2 Hz)	21.1	3.02 (*dd*, *J* = 7.4; 14.2 Hz)<br>3.10 (*dd*, J = 7.4 ; 14.2 Hz)	20.6
8	5.12 (*t*, *J* = 7.2 ; 7.2 Hz)	121.3	5.12 (*t*, *J* = 7.2 ; 7.2 Hz)	120.9
9		132.6		132.8
10	1.69 (*s*)	26.0	1.69 (*s*)	26.3
11	1.75 (*s*)	17.7	1.73 (*s*)	17.4
12	2.43 (*dd*, *J* = 7.8 ; 14.0 Hz)<br>2.54 (*dd*, *J* = 7.8 ; 14.0 Hz	42.6	2.43 (*dd*, *J* = 7.8 ; 14.0 Hz)<br>2.56 (*dd*, *J* = 7.8 ; 14.0 Hz)	42.5
13	5.00 (*t*, *J* = 7.9 ; 7.9 Hz)	115.7	5.02 (*t*, *J* = 7.8 ; 7.8 Hz)	115.7
14		138.2		138.5
15	1.68 (*s*)	25.7	1.68 (*s*)	25.7
16	1.52 (*s*)	17.8	1.52 (*s*)	17.6
1'		200.4		204.9
2'	2.77 (*q*, *J* = 6.7; 14.0 Hz)	46.2	3.72 (*m*)	34.4
3'	2.17 (*m*)	26.4	1.19 (*d*, *J* = 6.8 Hz)	18.9
4'	1.00 (*d*, *J* = 6.7 Hz)	22.8	1.12 (*d*, *J* = 6.8 Hz)	18.1
5'	0.96 (*d*, *J* = 6.7 Hz)	22.5		

Assignments were also established by COSY, HSQC-DEPT, HMBC

[a] 500 MHz, chemical shifts in ppm relative to TMS, ^{3}J in Hz; *s:* singulet, *d:* doublet, *dd:* dedoubled doublet, *q:* quadruplet, *t:* triplet, *m:* multiplet

Table S4. ^{1}H and ^{13}C NMR data for lupulone and colupulone in $CDCl_3$[a]

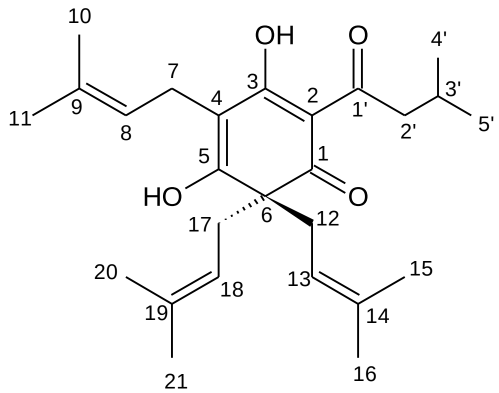

Lupulone

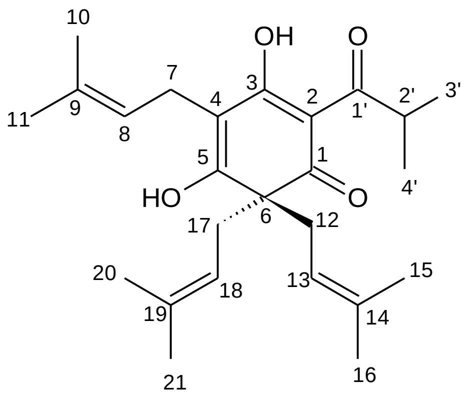

Colupulone

	β-acids			
	**Lupulone**		**Colupulone**	
Position	δ ^{1}H	δ ^{13}C	δ ^{1}H	δ ^{13}C
1		196.1		195.5
2		108.8		110.1
3		190.4		190.5
4		110.1		109.9
5		172.8		172.9
6		57.0		57.3
7	3.17 (*q*, *J* = 7.3 ; 14.2 Hz)	21.1	3.17 (*q*, *J* = 7.3 ; 14.2 Hz)	20.7
8	5.12 (*t*, *J* = 7.5 ; 7.0 Hz)	121.5	5.12 (*t*, *J* = 7.5 ; 7.0 Hz)	121.1
9		137.8		137.4
10	1.76 (*s*)	25.7	1.76 (*s*)	25.3
11	1.77 (*s*)	18.7	1.77 (*s*)	17.3
12	2.48 (*dd*, *J* = 7.8 ; 13.8 Hz)<br>2.63 (*m*)	37.9	2.47 (*dd*, *J* = 7.8 ; 13.8 Hz)<br>2.65 (*m*)	37.2
13	4.77 (*t*, *J* = 7.7 ; 7.7 Hz)	118.3	4.79 (*t*, *J* = 7.7 ; 7.7 Hz)	118.4
14		135.1		135.1
15	1.56 (*s*)	25.8	1.56 (*s*)	25.3
16	1.54 (*s*)	17.8	1.54 (*s*)	17.3
17	2.48 (*dd*, J = 7.8 ; 13.8 Hz)<br>2.63 (*m*)	37.9	2.47 (*dd*, J = 7.8 ; 13.8 Hz)<br>2.65 (*m*)	36.8
18	4.77 (*t*, *J* = 7.6 ; 7.6 Hz)	118.3	4.79 (*t*, *J* = 7.6 ; 7.6 Hz)	117.9
19		135.1		135.1
20	1.56 (*s*)	25.8	1.56 (*s*)	25.3
21	1.54 (*s*)	17.8	1.54 (*s*)	17.3
1'		202.2		207.3
2'	2.89 (*m*)	48.1	4 (*m*)	35
3'	2.1 (*m*)	26.1	1.10 (*d*, *J* = 6.8 Hz)	18.1
4'	0.94 (*d*, *J* = 6.5 Hz)	22.8	1.10 (*d*, *J* = 6.8 Hz)	18.1
5'	0.93 (*d*, *J* = 6.5 Hz)	22.8	-	-

Assignments were also established by COSY, HSQC-DEPT, HMBC

[a] 500 MHz, chemical shifts in ppm relative to TMS, ^{3}J in Hz; *s:* singulet, *d:* doublet, *dd*: dedoubled doublet, *t:* triplet, *q:* quadruplet, *m:* multiplet

Figure S1. Chromatograms at 370 nm of the crude hydro-ethanolic extract of cones and purified chalcones (desmethylxanthohumol and xanthohumol)

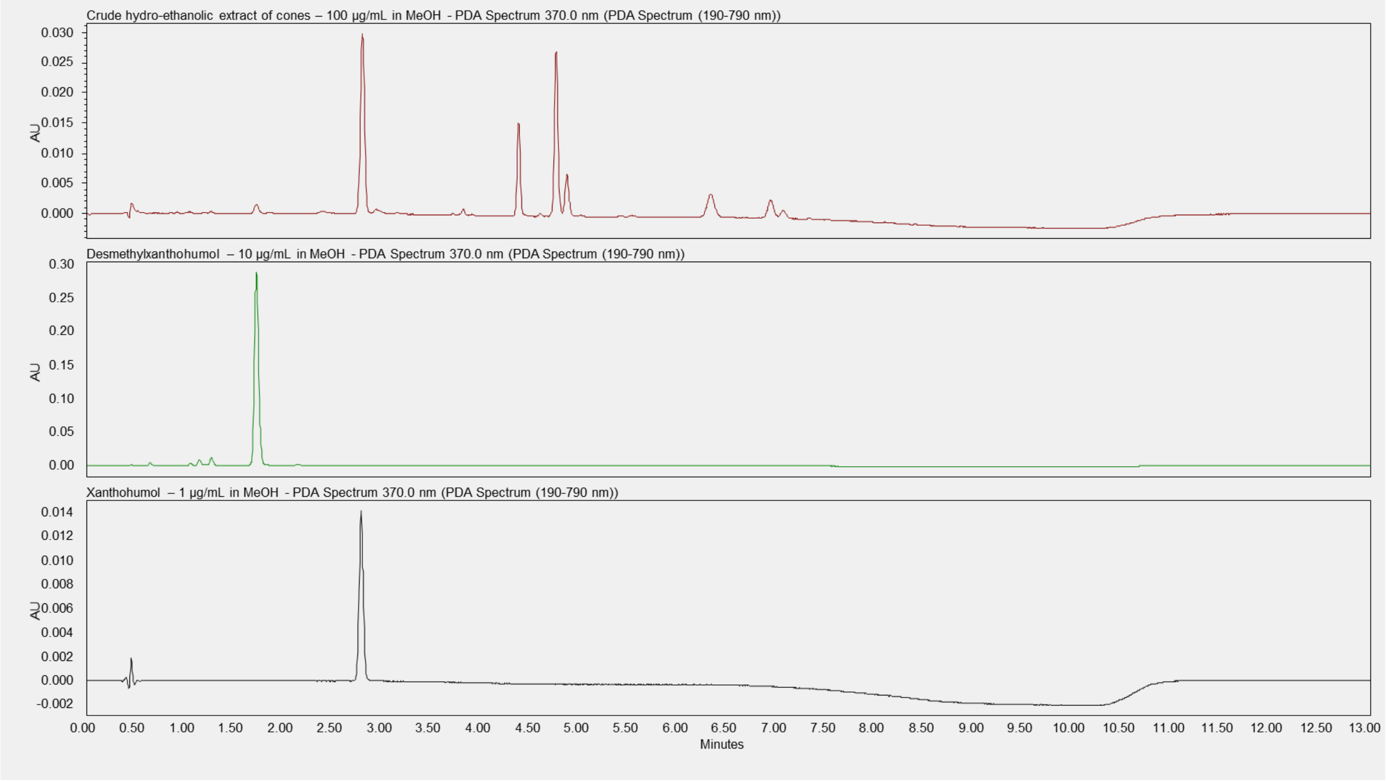

Figure S2. Chromatograms at 330 nm of the crude hydro-ethanolic extract of cones and purified acylphloroglucinol derivatives (cohumulone, humulone, colupulone and lupulone)

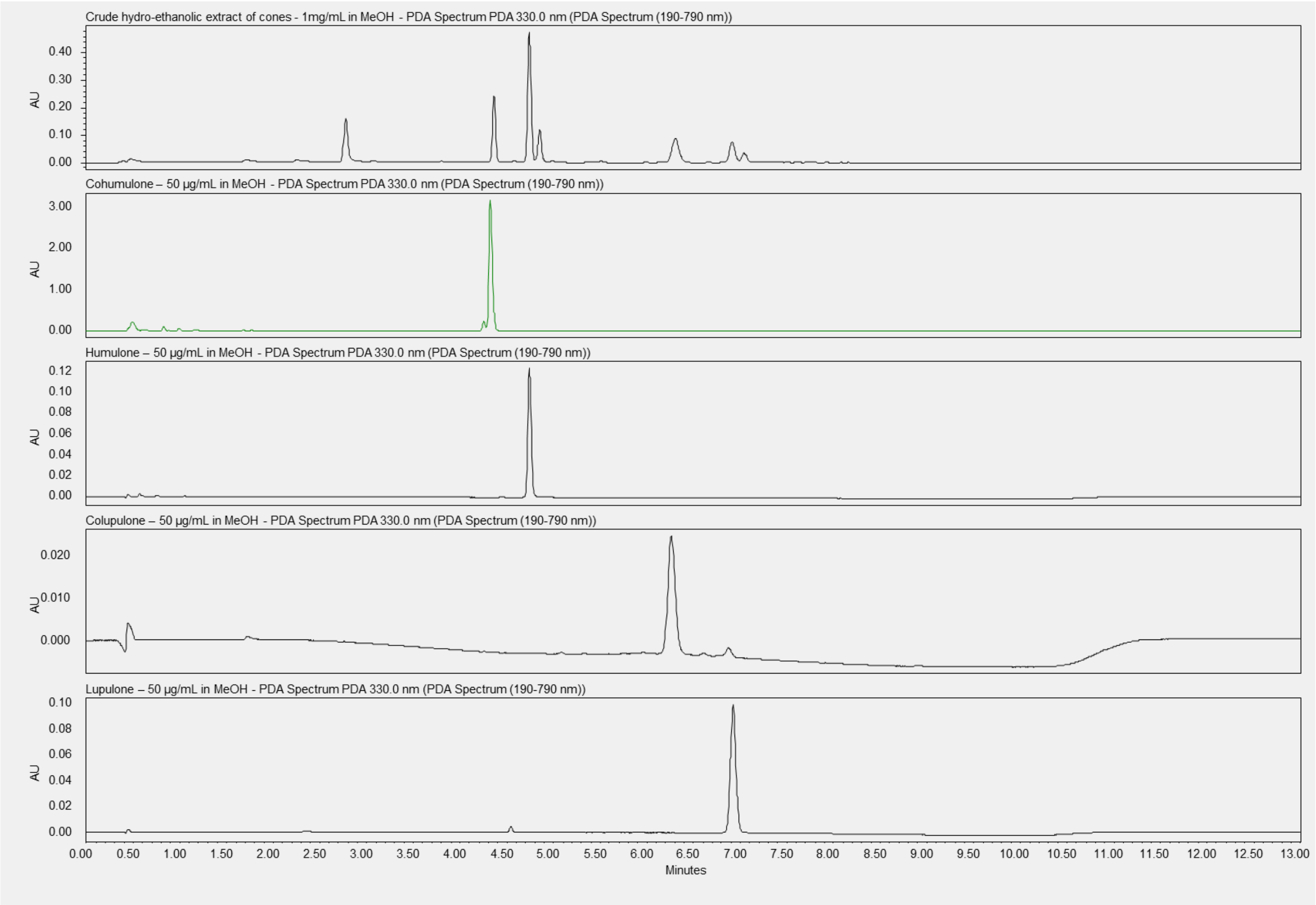

Figure S3. Total ion chromatogram of the crude hydro-ethanolic extract of cones in negative mode and selected ion recording of purified chalcones (desmethylxanthohumol and xanthohumol) and acylphloroglucinol derivatives (cohumulone, humulone, colupulone and lupulone)

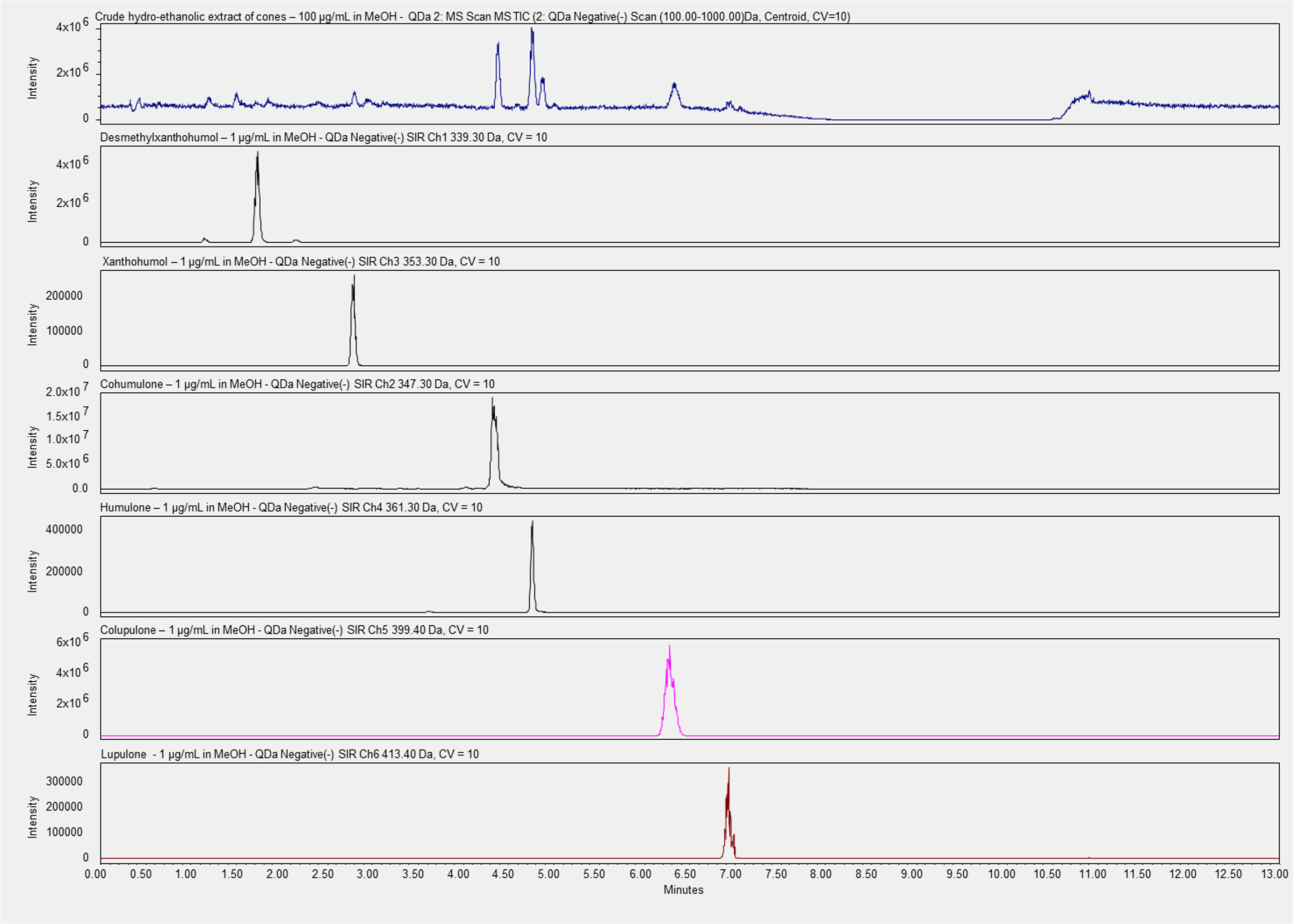

Supplement: Supplementary file 1 [file molecules-24-01024-s001.pdf]
